# Estimating Species Richness and Modelling Habitat Preferences of Tropical Forest Mammals from Camera Trap Data

**DOI:** 10.1371/journal.pone.0103300

**Published:** 2014-07-23

**Authors:** Francesco Rovero, Emanuel Martin, Melissa Rosa, Jorge A. Ahumada, Daniel Spitale

**Affiliations:** 1 Tropical Biodiversity Section, MUSE - Museo delle Scienze, Trento, Italy; 2 Udzungwa Ecological Monitoring Centre, Udzungwa Mountains National Park, Mang’ula, Tanzania; 3 Sokoine University of Agriculture, Morogoro, Tanzania; 4 Tropical Ecology Assessment & Monitoring (TEAM) Network, Betty & Gordon Moore Center for Science and Oceans, Conservation International, Washington, D.C., United States of America; Università degli Studi di Napoli Federico II, Italy

## Abstract

Medium-to-large mammals within tropical forests represent a rich and functionally diversified component of this biome; however, they continue to be threatened by hunting and habitat loss. Assessing these communities implies studying species’ richness and composition, and determining a state variable of species abundance in order to infer changes in species distribution and habitat associations. The Tropical Ecology, Assessment and Monitoring (TEAM) network fills a chronic gap in standardized data collection by implementing a systematic monitoring framework of biodiversity, including mammal communities, across several sites. In this study, we used TEAM camera trap data collected in the Udzungwa Mountains of Tanzania, an area of exceptional importance for mammal diversity, to propose an example of a baseline assessment of species’ occupancy. We used 60 camera trap locations and cumulated 1,818 camera days in 2009. Sampling yielded 10,647 images of 26 species of mammals. We estimated that a minimum of 32 species are in fact present, matching available knowledge from other sources. Estimated species richness at camera sites did not vary with a suite of habitat covariates derived from remote sensing, however the detection probability varied with functional guilds, with herbivores being more detectable than other guilds. Species-specific occupancy modelling revealed novel ecological knowledge for the 11 most detected species, highlighting patterns such as ‘montane forest dwellers’, e.g. the endemic Sanje mangabey (*Cercocebus sanjei*), and ‘lowland forest dwellers’, e.g. suni antelope (*Neotragus moschatus*). Our results show that the analysis of camera trap data with account for imperfect detection can provide a solid ecological assessment of mammal communities that can be systematically replicated across sites.

## Introduction

Profiling large-bodied animal communities, such as mammals, fundamentally implies assessing species richness and composition. Determining a state variable of species’ abundance is also required to make inferences on species distribution, habitat associations, and trends over time [1]–[4]. In this context, medium-to-large mammals in tropical forests are of priority because they represent a rich and functionally diversified component of this biome, and yet they are universally threatened by hunting, and habitat loss and fragmentation [5]–[8]. The removal, or decrease in abundance, of tropical mammals will likely impact forest dynamics [11], [12] due to their direct involvement in seed predation, seed dispersal, herbivore control, nutrient cycling and other ecosystem functions [9], [10]. Systematic assessments that allow inference of tropical forest mammal communities in space and time remain limited and a chronic gap persists in standardized data collection.

The Tropical Ecology, Assessment and Monitoring (TEAM) network was set-up to fill this gap by establishing a network of field stations, scientists and partners across the tropics for long-term monitoring of mammal communities using a standardized and annually repeated sampling protocol [13]. The excellent potential of TEAM network data for answering questions on the status and trends of mammals has already been shown both through the first pan-tropical analysis from seven sites, which compared communities’ richness and composition against forest area and fragmentation [8], as well as the first assessment of temporal changes at one particular site in Costa Rica using dynamic occupancy analysis [14]. In the present study, we used data from the first TEAM site established in Africa in 2009, the Udzungwa Mountains of south-central Tanzania, to propose a standardized approach for assessing the community of medium-to-large mammals detected through camera-trapping during the first, baseline year of the long-term programme.

The use of camera trapping for wildlife studies has increased exponentially in the last decade as it is an efficient, cost-effective and easily replicable tool to study and monitor ground-dwelling terrestrial mammals and birds [15], [16]. Camera trapping is particularly suited to collect standardized data because sampling effort can be easily controlled for and sampling design can be replicated across time and space [15]. In addition, sampling can be considered as multiple occasions during a discrete season, hence data are suited for analyses that account for imperfect detection, such as occupancy [17], [18]. Occupancy (*ψ*) is defined as the proportion of area, patches or sites occupied by a species [19] and can be used as a surrogate for abundance [20]. Detection probability (*p*) is defined as the likelihood of detecting an individual, or species, during a sampling occasion [18]. With the inclusion of covariates, occupancy models provide a robust statistical framework for testing scientific hypotheses. For example, one can test for differences in occupancy rates between study sites that contrast by habitat type, hunting level, distance to key resources, climate conditions and vegetation features [21], [22]. In addition, the same approach used for occupancy analysis can also be used for estimating species richness and accumulation [23].

The Udzungwa Mountains are an area of outstanding importance for biodiversity endemism and conservation in Africa [24], and are particularly rich in forest dwelling mammals [25]. Through our assessment we aimed to (1) evaluate sampling effort and estimate species’ richness, (2) determine drivers of variation in species richness and detection probability (*p*) using an occupancy framework [17], (3) estimate species’ occupancy (*ψ*), and (4) determine the best habitat and human disturbance predictors of both *ψ* and *p* to identify major patterns of species’ responses to these predictors.

## Materials and Methods

### Ethics Statement

Data collection used non-invasive, remotely set camera traps and hence did not involve direct contact or interaction with the animals. Fieldwork was done under research permit number 2009-139-NA-2009-49 to FR, issued by the Tanzania Commission for Science and Technology (COSTECH).

### Study area

**Figure 1:**
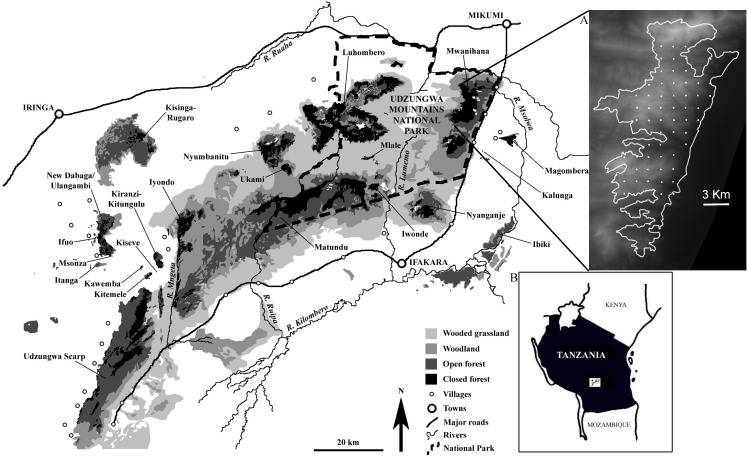
Map of the study area, the Udzungwa Mountains of south-central Tanzania. The map shows the main habitat types and blocks with closed-canopy forest (adapted from [53]). The study forest was Mwanihana in the northeastern portion of the range, which is zoomed in inset (**A**) where the 60 camera trap sites are shown as white dots and the background is a Digital Elevation Model (dark is lower elevation); (**B**) shows the position of Udzungwa in Tanzania.

The Udzungwa Mountains of south-central Tanzania (over 10,000 km^2^; 7°40′–8°40′S, 35°10′–36°50′E) are a mosaic of moist forest blocks interspersed with drier habitats. The study was conducted in Mwanihana forest, which at 180 km^2^ is one of the largest forests in the area and with the widest, continuous forest elevation range (290–2250 m a.s.l.; Fig. 1). The forest is inside the Udzungwa Mountains National Park (1990 km^2^). The eastern border of the forest coincides with the eastern boundary of the park. The forest habitat broadly ranges east-west from lowland, deciduous forest to montane, evergreen forest [26]. The lower elevation habitats, which include deciduous, semi-deciduous and riverine evergreen forest, have been degraded historically and have large portions of secondary, regenerating vegetation. The interior forest is mainly undisturbed with large chunks of pristine, closed-canopy moist forest. Anthropogenic disturbance in the form of firewood collection occurred at the lower elevations, a practice likely coupled with illegal bush meat hunting done using snares. The upper elevation zone has lower canopy and bamboo forest with rocky and very steep areas, especially in the northern part. Total rainfall in Mwanihana forest is around 1500 mm per year (data from Udzungwa Mountains National Park); rainfall measured at 1200 m a.s.l. by an automatic rainfall gauge was 1387 and 1451 mm in 2011 and 2012, respectively (FR/TEAM Network, unpublished data). The dry season spans from June to November, while two rainy seasons occur during November-June. In 2012, mean monthly air temperature at 1200 m a.s.l. ranged 17.2–22.6°C. (FR/TEAM Network, unpublished data).

### Data collection

Camera trapping was conducted from July to November 2009, as the baseline year of the TEAM programme [13]. We used digital cameras (Reconyx RM45, Reconyx Inc., Holmen, Wisconsin, USA) set to take photos without delay between consecutive triggers. Using ESRI’s ArcGIS 10 software, we designed a regular grid of 60 camera trap locations at a density of one camera per 2 km^2^, and placed at random across the forest. We then conducted a ground survey to select the final camera positions, and locations that fell in excessively steep, open canopy or rocky areas were repositioned no more than 100 m from the original location (Fig. 1).

Camera traps were positioned so the field of view included an active wildlife trail and then secured to a tree about 2–3 m away from the trail at an average height of 50 cm and left running for 30 days. Since cameras can run automatically over such period, we did not check them to avoid unnecessary disturbance. Due to limits in the number of cameras available and the time needed for the field team to set cameras, we sampled the 60 points by deploying three consecutive arrays of 20 camera traps (south, central and northern Mwanihana, respectively). Therefore, the data collection lasted 133 days from July 24 to December 4, 2009.

At sampling completion, memory cards were recovered and images were identified using specialized software (DeskTEAM, [27]; see also www.teamnetwork.org/en/help-deskteam). A single taxonomic authority [28] was used across all TEAM sites for species identification, except for species that were not included in this reference or species that were re-assessed. The validated and publicly available data were downloaded from the TEAM portal (data package id: TV-20111116005138_3515).

### Data analysis

We derived standard descriptors of mammal community by filtering the image records for each species of mammal to derive the number of events per hour, hence avoiding that multiple images of the same individual pausing in front of the camera trap were scored as multiple events [29]. We then computed a relative abundance index (RAI) as the number of events divided by sampling effort and multiplied by 100 (i.e. events per 100 days of camera trapping). We also computed the naïve occupancy as the number of camera trap sites occupied on sites sampled.

We derived a number of spatial environmental covariates deemed relevant to explain both the spatial variations of species’ richness and occupancy of selected species using geoprocessing tools available in ArcGIS. We calculated the following variables: (1) distance from eastern park border (‘border’), (2) distance from forest edge (‘edge’), (3) forest habitat type, i.e. montane forest and lowland forest (‘habitat’), (4) slope and (5) distance to rivers. The distance from each camera trap point to the nearest ‘border’, ‘edge’, or river segment was calculated in ArcGIS. Forest habitat type was mapped using a supervised classification approach on Landsat TM and ETM+ satellite imagery (30 m resolution). Habitats were categorized into three forest types: 1) Montane, 2) Deciduous, and 3) Regenerating. Forest habitat type was then extracted for each camera trap point in ArcGIS. ‘Border’ correlated highly with elevation at camera trap sites given the forest morphology of an east-west escarpment (Pearson’s *r* = 0.802, *P*<0.001) and it is considered a proxy of decreasing anthropogenic disturbance, which may be mainly associated to firewood collection and pole/timber cutting [30]. After checking that no collinearity existed among the covariates used, these were standardized to have mean 0 and unit variance before estimating the model coefficients.

As a fundamental measure of the community structure, we analysed species richness under three different perspectives and with different aims. (1) Species accumulation curve with cumulative camera trap days was used to check if data collection lasted a sufficient number of days to virtually capture the total number of species. The order in which samples (they consisted of number of events per day) were included in the curve was randomized 1000 times and results were used to derive 95% confidence intervals around the mean [31]. Even though this approach ignores imperfect detection of individual species, it is useful for comparison with other studies [32], [33]. (2) Analysis of species richness that accounts for imperfect detection was studied using the model by Dorazio and colleagues [23], which requires repeated temporal replications to resolve the ambiguity between species absence and non-detection when species are unobserved at sample locations. This Bayesian approach combines community-level and species-level attributes in the same framework, allowing either community-level or species-level parameters to be evaluated. Such flexibility is not matched by other methods for estimating species richness [23]. The frequentist approach to the same problem is possible, but computationally intensive to implement [23]. The model was specified in BUGS language and fitted to data using WinBUGS and the package ‘R2WinBUGS’ in R software [34], [35]. Simulations were executed with five Markov chains; 55,000 iterations for each chain, discarding 5,000 iterations at the beginning (burn-in) and setting the thinning rate to 50. This returned 5,000 samples from the posterior distributions. (3) We used the occupancy analysis framework to investigate possible relationships between species richness and environmental covariates [19], [36]. In particular, we compared two sets of models: (1) testing the effect of environmental covariates on the occupancy of all the species (species richness), and (2) testing if trophic guild (carnivores, herbivores, omnivores, insectivores) and body mass (data from [37]) were related to detection probability. Akaike Information Criterion (AIC) was used to rank all the candidate models and calculate their Akaike weights [38]. To achieve intra-guild homogeneity, we discarded elephant (*Loxodonta africana*) and buffalo (*Syncerus caffer*) among the herbivores for their large body mass and movement habits (i.e. they periodically move into the forests from drier habitats in the park). Among the carnivores, the bushy-tailed mongoose (*Bdeogale crassicauda*) was discarded because it is a common, non-elusive, and partially omnivorous species; hence it effectively represents an outlier in the carnivore guild.

We also used occupancy [17] as the species-specific state variable of abundance to assess differences across species under an unbiased framework and determine covariates of both occupancy and detection probability for a set of species. We used scripts already developed [8] and implemented in R to arrange the TEAM data (http://www.teamnetwork.org/) into a list of species’ occupancy matrices. Data for each species were arranged as matrices of sites by surveys (i.e. sampling occasion). Each entry indicated if the species was observed at site *i* on survey *j* or not. If the species was observed at site *i* on survey *j*, then the entry was given a score of 1. If the species was not observed, then the entry was given a score of 0. NA indicated site *i* was not sampled on survey *j*. The species-specific occupancy matrix had a resolution of five days.

We used these matrices as the input for the single-season occupancy model [19]. We modelled both estimated occupancy (*Ψ*) and detection probability (*p*) with and without covariates. A common set of models was used for all the species. In addition to the null model, that assumes constant *Ψ* and *p* (i.e. *Ψ*(.), *p*(.)), for other models *p* was allowed to vary by distance to border and distance to edge. In both cases, our hypothesis was that animals would be more elusive near the border and/or the edge because of greater disturbance [30]. Four covariates for *Ψ* were the following: (1) ‘border’, (2) ‘edge’, (3) ‘river’ and (4) ‘habitat’. Numerical covariate were standardized into z-scores and included both individually and in combination. We used the Akaike Information Criterion (AIC) to rank candidate models and calculate their Akaike weights [38]. In the case of top-ranked models with similar AIC (and weight >0.01), we applied a model-averaging technique to estimate occupancy from these multiple models [38]. Occupancy analysis was performed using the package ‘unmarked’ in R [39]. The relative importance of the model parameters were calculated with the R package AICmodavg [40]. Once we identified the best occupancy model (or the average of the best models), we mapped occupancy probability across Mwanihana forest by deriving occupancy estimates from covariates computed on a spatial grid with a cell size of 100 m.

## Results

**Figure 2:**
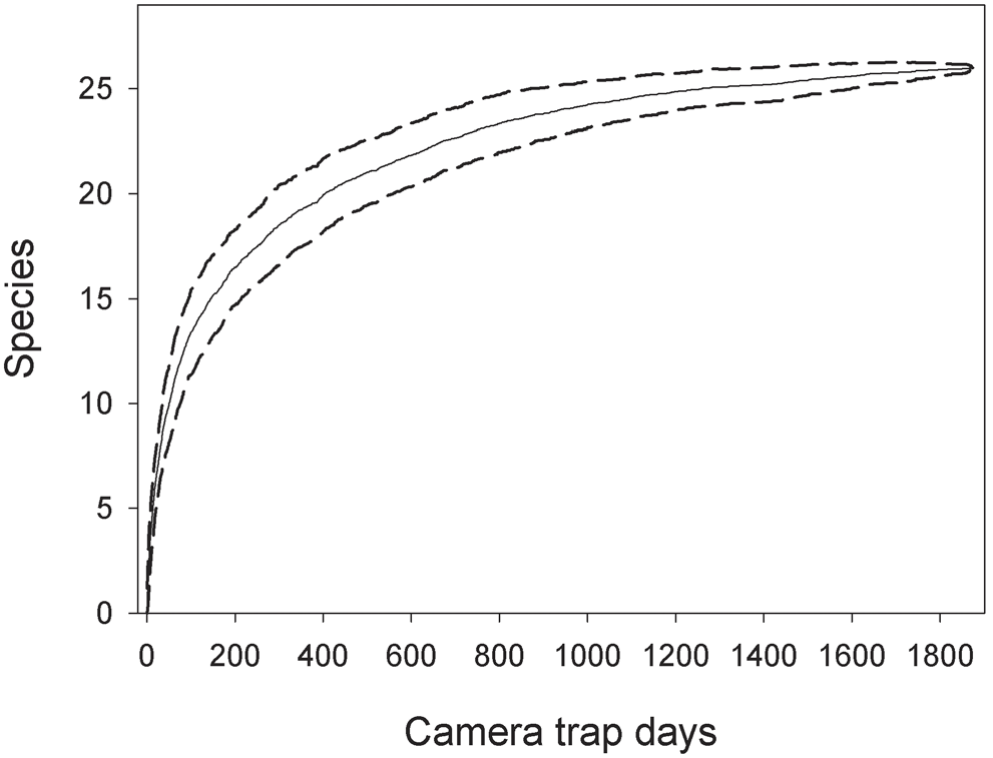
Species accumulation curve for the community of medium-to-large mammals detected by camera trapping. Detection of species is randomized 1000 times and results used to derive the 95% confidence intervals of the mean.

**Figure 3:**
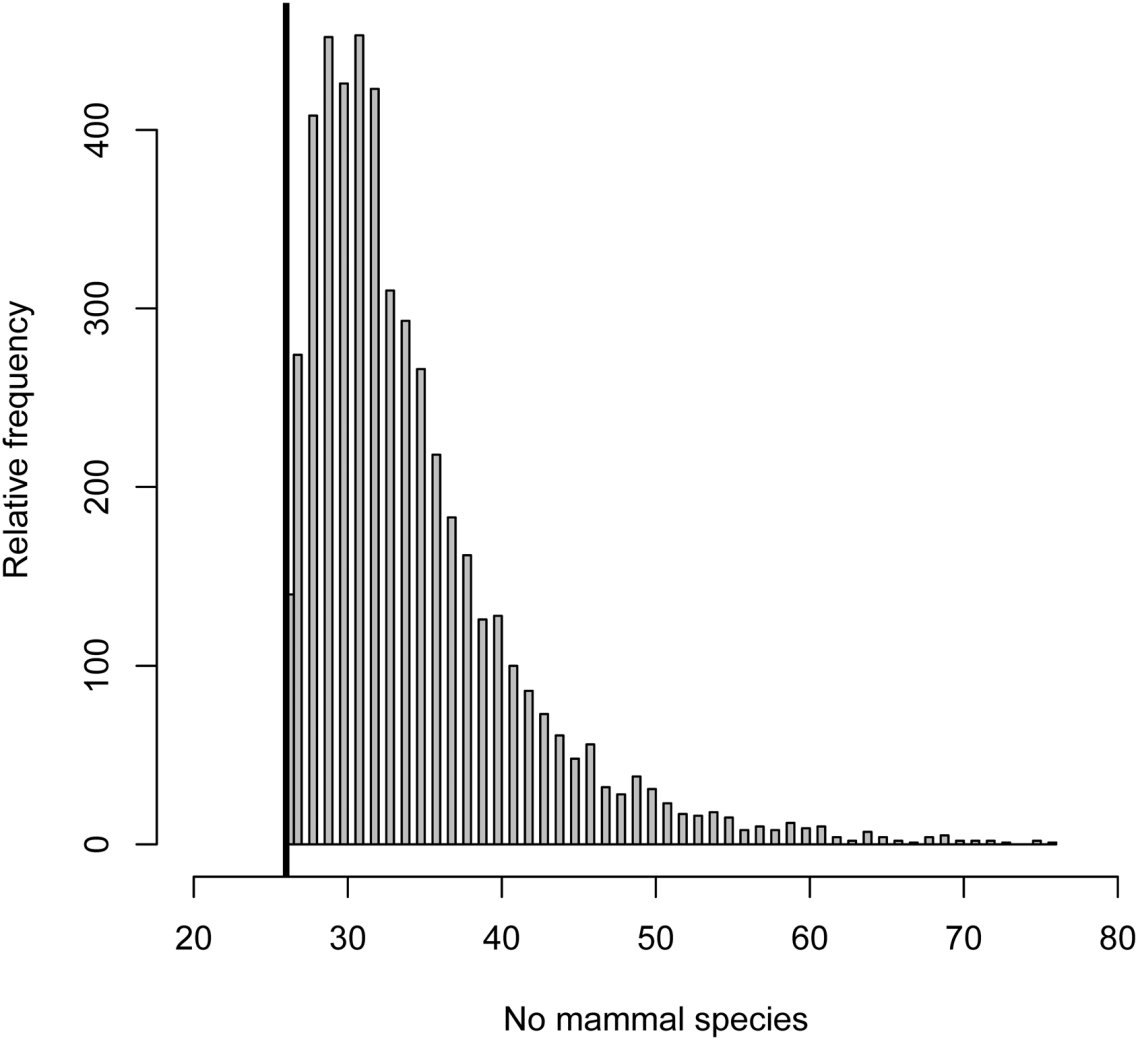
Posterior distribution of species richness. The analysis follows Dorazio et al. (2006). The posterior probability that the community comprises only 26 species (vertical line is the observed species richness) is essentially zero, and the estimated median and mean values of species richness are 32.0 (±7.04 SD) and 34.3, respectively (26–54 CRI 95%; CRI = credible intervals).

Of the 60 camera traps set, two malfunctioned and the remaining 58 accumulated 1,818 camera days (mean 31.34) yielding 10,647 images of mammals. The list of 26 species recorded and standard descriptors are reported in Table 1. The range of species captured per camera was 3–10 (median 6). Four species were recorded with >100 events in this order: (1) Harvey’s duiker (*Cephalophus harveyi*), (2) giant-pouched rat (*Cricetomys gambianus*), (3) bushy-tailed mongoose and (4) suni. Six species scored >20 and ≤100 events: (1) Abbott’s duiker (*Cephalophus spadix*), (2) Tanganyika mountain squirrel (*Paraxerus vexillarius*), (3) grey-faced sengi (*Rhynchocyon udzungwensis*), (4) Sykes’ monkey (*Cercopithecus mitis*) and (5) tree hyrax (*Dendrohyrax validus*). The remaining 16 species scored ≤20 events, of which 10 species scored ≤5 events. The accumulation of species detected with sampling effort was initially steep, but by 1,000 camera days the majority of species were detected (24 species, or 92%; Fig. 2). The estimated size of the community according to [23] exceeds the number of species observed in the sample by a substantial margin, with median and mean values being 32 and 34.3, respectively (Fig. 3).

**Table 1:** List of mammals camera trapped in Mwanihana forest, Udzungwa Mountains, Tanzania, reporting species traits (body mass from Smith et al. (2003) and functional guild from IUCN (2013)) and three raw indices of abundance: hourly events, relative abundance index (RAI) and naïve occupancy (number of sites that are positive to species’ presence divided by the total number of sites sampled).

#	Taxonomic group	Common name	Latin name	Mass (kg)	Functional guild	Events per hour	RAI (events/100 days)	Naïve occupancy
1	Afrotheria	Tree hyrax	*Dendrohyrax arboreus*	2.95	Omniv	23	1.27	0.241
2		African elephant	*Loxodonta africana*	3900	Herbiv	11	0.61	0.121
3		Four-toad sengi	*Petrodromus tetradactylus*	0.19	Insectiv	3	0.17	0.017
4		Chequered sengi	*Rhynchocyon cirnei*	0.49	Insectiv	4	0.22	0.052
5		Grey-faced sengi	*Rhynchocyon udzungwensis*	0.80	Insectiv	45	2.48	0.259
6	Carnivores	Marsh mongoose	*Atilax paludinosus*	3.30	Carniv	3	0.17	0.052
7		Bushy-tailed mongoose	*Bdeogale crassicauda*	1.55	Carniv	130	7.15	0.741
8		African civet	*Civettictis civetta*	12	Omniv	1	0.06	0.017
9		Lowe’s servaline genet	*Genetta servalina lowei*	1.06	Omniv	18	0.99	0.259
10		Honey badger	*Mellivora capensis*	8.50	Carniv	7	0.39	0.103
11		Banded mongoose	*Mungos mungo*	1.93	Insectiv	2	0.11	0.034
12		African palm civet	*Nandinia binotata*	1.90	Carniv	2	0.11	0.034
13		Leopard	*Panthera pardus*	52	Carniv	8	0.44	0.052
14	Primates	Sanje mangabey	*Cercocebus sanjei*	8	Omniv	73	4.02	0.517
15		Sykes’ monkey	*Cercopithecus mitis*	5	Omniv	22	1.21	0.241
16		Yellow baboon	*Papio cynocephalus*	18.4	Omniv	3	0.17	0.052
17		Udzungwa red colobus	*Procolobus gordonorum*	10	Omniv	5	0.28	0.069
18		Angolan colobus	*Colobus angolensis*	8.6	Omniv	1	0.06	0.017
19	Rodents	Giant pouched-rat	*Cricetomys gambianus*	1.24	Omniv	276	15.18	0.534
20		Cape porcupine	*Hystrix africaeaustralis*	14.94	Omniv	11	0.61	0.086
21		Tanganyika mountain squirrel	*Paraxerus vexillarius*	0.68	Herbiv	46	2.53	0.328
22	Ungulates	Harvey’s duiker	*Cephalophus harveyi*	12	Herbiv	367	20.19	0.862
23		Abbott’s duiker	*Cephalophus spadix*	56	Herbiv	60	3.30	0.466
24		Suni	*Neotragus moschatus*	6.5	Herbiv	114	6.27	0.448
25		Bush pig	*Potamochoerus larvatus*	48.78	Omniv	18	0.99	0.190
26		African buffalo	*Syncerus caffer*	580	Herbiv	4	0.22	0.052

Trophic guilds: Herbiv = herbivores, Omniv = omnivores, Insectiv = insectivores, Carniv = carnivores.

**Figure 4:**
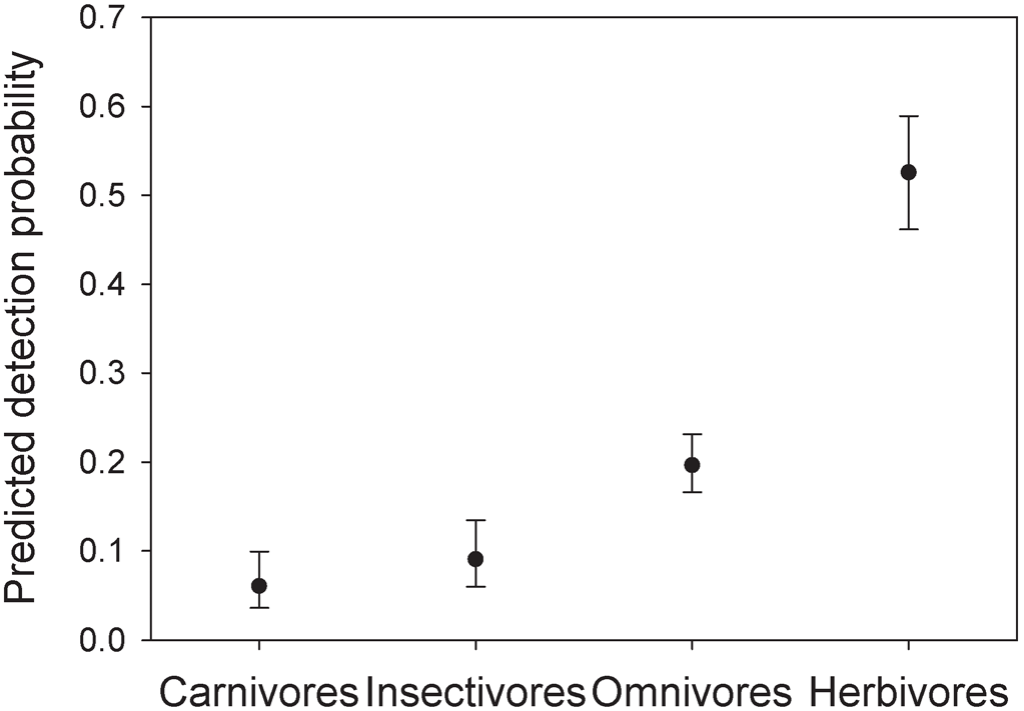
Detection probability by functional guild. Values are from the model averaging of relative species richness of the mammal community in the Udzungwa Mountains of Tanzania. Bars are 95% confidence intervals.

The modelling of species richness using a sub-set of 23 species revealed no support for the null model, with several models having lower AIC, and five that were top-ranked with delta AIC<3 (Table 2). Model averaging using these first five models shows that no environmental covariates affected relative species richness. However, there is a significant influence of the functional guild on the detection probability (Table 3). Herbivores had the highest detection probability (0.52±0.03 SE), followed by omnivores (0.20±0.02 SE), insectivores (0.09±0.02 SE) and carnivores (0.06±0.02 SE; Fig. 4).

**Table 2:** Summary of model selection outcome for predictions of mammal species’ richness in the Udzungwa Mountains of Tanzania.

Model	Number ofparameters	AIC	Delta	AIC weight	Cumulative weight
*ψ*(.) *p*(mass, guild)	6	1151.59	0.00	0.410	0.410
*ψ*(.) *p*(guild)	5	1152.57	0.98	0.250	0.660
*ψ*(habitat, species) *p*(mass, guild)	7	1153.59	2.00	0.150	0.810
*ψ*(border) *p*(guild)	6	1154.56	2.97	0.093	0.910
*ψ*(habitat) *p*(guild)	6	1154.56	2.98	0.093	1.000
*ψ*(.) *p*(.)	2	1323.63	172.04	0.000	1.000

The top-ranked models are shown (delta AIC <3) followed by the null model.

**Table 3:** Summary of model averaging for the effect of environmental covariates on species richness (*ψ*) and detection probability (*p*) of the mammal community in the Udzungwa Mountains of Tanzania.

Model	Estimate	SE	*Z*	*P*(>|z|)
*p*(mass)	–0.136	0.08	1.704	0.089
*p*(herbivores)	2.860	0.31	9.345	<0.001
*p*(insectivores)	0.380	0.37	1.040	0.298
*p*(omnivores)	1.327	0.30	4.497	<0.001
*ψ*(habitat - montane)	2.814	292.60	0.010	0.992
*ψ*(border)	1.273	628.75	0.002	0.998

See Table 2 for the covariates modelled with both *ψ* and *p*.

We could fit occupancy models for the 11 most recorded species. We initially considered 14 species with ≥10 events or naïve occupancy ≥0.1; however, for three of these (*Genetta servalina*, *Loxodonta africana*, *Hystrix africaeaustralis*) the models did not converge. For these 11 species, *ψ* ranged from 0.25–0.86 and *p* ranged from 0.10–0.51. The null model was not supported for any of these species, and at least one of the covariates considered affected significantly or marginally significantly *ψ* and *p* (Table 4). Details of model selection for each species are shown in Table S1.

**Table 4:** Summary of species-specific occupancy estimates for 11 mammals that had adequate detection for the analysis, ordered by decreasing estimated occupancy (*ψ*).

Species	Naïve *ψ*	*ψ*	SE(*ψ*)	*p*	SE(*p*)	*p*(border)	*p*(edge)	*ψ*(border)	*ψ*(edge)	*ψ*(river)	*ψ*(habitat [type])
*Cephalophus harveyi*	0.862	0.876	0.080	0.432	0.046	(+)			–	(–)	
*Bdeogale crassicauda*	0.741	0.815	0.069	0.298	0.039	–					
*Cephalophus spadix*	0.466	0.716	0.094	0.171	0.040	+	+				
*Cercocebus sanjei*	0.517	0.615	0.107	0.234	0.057						+[montane]
*Cricetomys gambianus*	0.534	0.539	0.118	0.505	0.033					+	
*Cercopithecus mitis*	0.241	0.503	0.198	0.096	0.039		–				
*Dendrohyrax validus*	0.241	0.480	0.116	0.106	0.039		(+)			+	
*Neotragus moschatus*	0.448	0.472	0.105	0.374	0.057		+	(+)			–[montane]
*Paraxerus vexillarius*	0.328	0.400	0.100	0.203	0.040				+		
*Potamochoerus larvatus*	0.190	0.387	0.150	0.098	0.055	(–)					
*Rhynchocyon udzungwensis*	0.259	0.278	0.114	0.298	0.048				(+)		(+) [montane]

This is the average value of predicted occupancy at the 58 camera trap localities from the final models. Significant outcomes of the effects of covariates on *ψ* and detection probability (*p*) are also indicated with their directionality (positive/negative effect), which is in parenthesis when the significance is marginal (0.05<*P*<0.1). Naïve occupancy values are also shown to appreciate the differences with *ψ*, which are particularly remarkable for species with *p*<0.2.

**Figure 5:**
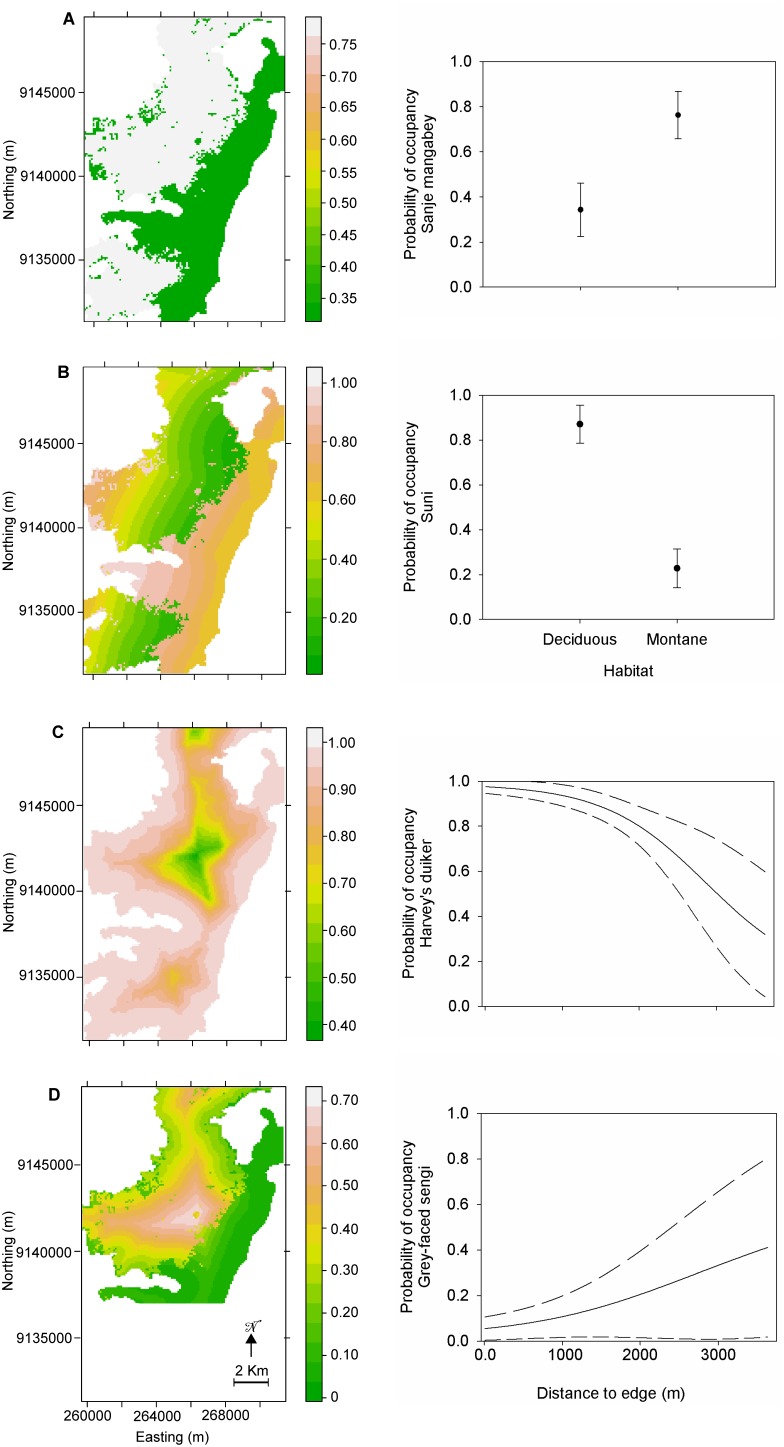
Spatially-explicit occupancy models. Maps of predicted occupancy (left) and functional relationship between the most relevant covariate and *ψ* (right, with confidence intervals indicated by dashed lines) for four mammals in the Udzungwa Mountains of Tanzania, representing limiting cases in occupancy pattern: (**A**) Sanje mangabey, a montane evergreen forest species; (**B**) suni, a lowland deciduous forest species; (**C**) Harvey’s duiker, an edge-lover and disturbance-tolerant species; (**D**) grey-faced sengi, an edge-avoider and disturbance-sensitive species.

The main patterns of predicted *ψ* and the functional relationships of *ψ* with the dominant covariate represented by the four species shown in Fig. 5 are as follows:

(1) As a ‘montane forest dweller’, Sanje mangabey’s *ψ* is positively associated with montane forest habitat and not affected by any of the other variables. Hence, predicted occupancy falls in two values of 0.34±0.12 SE in lowland, deciduous forest and 0.76±0.10 SE in montane, evergreen forest.
(2) In contrast with the above, suni is a ‘lowland forest dweller’, with *ψ* being negatively related to montane forest habitat; hence, indicating preference for lowland forest. In addition, the species’ *ψ* is marginally affected by distance to park border, with predicted occupancy decreasing in the proximity of park border relative to more interior zones of lowland forest. Its detection probability also significantly increased with distance to edge.
(3) The Harvey’s duiker is a typical ‘edge lover’ species, as *ψ* is negatively affected by distance to edge, which is clearly seen in the spatially-explicit model. Therefore, the species avoids interior forest, with predicted *ψ* declining sharply and non-linearly after 1.5–2 km from the forest edge.
(4) An opposite pattern is shown by the grey-faced sengi, which seems to be an ‘edge avoider’ with *ψ* being positively affected by distance from edge and preference for montane habitat and both associations being marginally significant (0.05<*P*<0.1).

**Figure 6:**
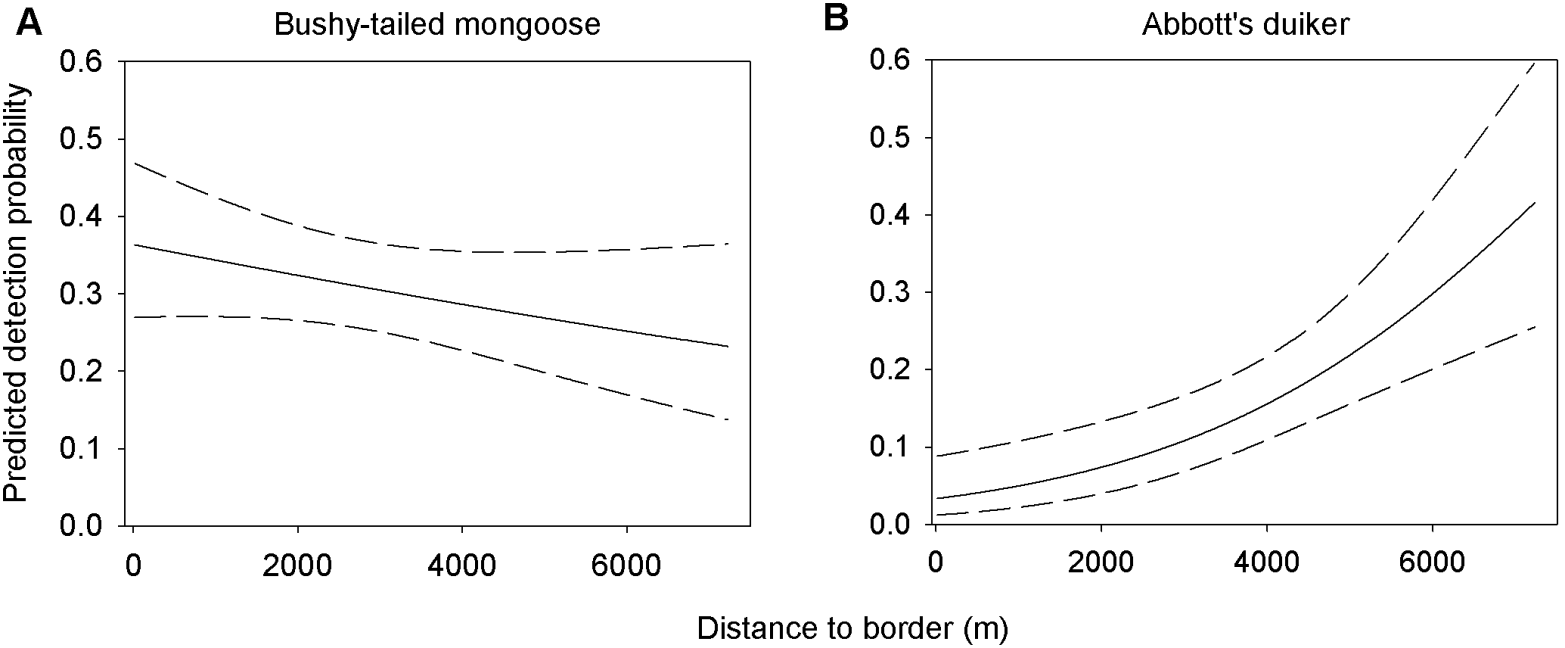
Graphs of predicted detection probability. Values are modelled with distance from the park border for (**A**) the bushy-tailed mongoose, and (**B**) the Abbott’s duiker, in the Udzungwa Mountains of Tanzania. Confidence intervals are indicated by dashed lines.

For approximately half of the species, detection probability varied with distance to the park border and/or distance to the forest edge (Online Resource 1). We portray two limiting cases (Fig. 6): (1) the bushy-tailed mongoose, where *p* decreased linearly with the distance to park border, and (2) the Abbott’s duiker, where *p* exponentially increased with distance to border.

## Discussion

Our study shows how camera trap data collected using a robust, standardized field methodology, and analysed with statistical approaches that account for imperfect detection and incorporate ecological factors, can provide a robust baseline assessment of mammal communities in tropical forests. In particular, we estimated species richness using a sampling protocol requiring repeated observations at sample locations. This approach provided information needed to resolve the ambiguity between species absence and non-detection. The Bayesian analysis used [23] is a flexible alternative to the classic frequentist approach, which is computationally complex, and combines community-level and species-level attributes in the same modelling framework. We also determined how simple ecological covariates such as gross habitat, distance to forest edge and distance to park border explain the occupancy of most species in the community. Finally, we provided a framework for deriving spatially-explicit, fine resolution models of estimated species occupancy in relation to covariates, which represent a valuable tool for conservation management of threatened and/or poorly known species.

The efficiency of camera trapping for inventorying species has already been indicated by other studies of tropical mammal communities [33], [41]. In the Udzungwas, additional camera trapping effort and scattered sighting reports indicate that at least four species have been ‘missed’ by the present survey (bushbuck *Tragelaphus scriptus*, spotted hyena *Crocuta crocuta*, cane rat *Thryonomys swinderianus* and serval cat *Leptailurus serval*; FR unpublished data). Other small, elusive carnivores may also be present in the target forest [42]. This observation is supported by our models, which estimate that >30 species occur. It is worth noting that the classic species richness estimators, parametric and non-parametric, asymptotic and non-asymptotic, rely on extrapolations of the species accumulation curve and do not account for imperfect detection [31], [43]. The explicit incorporation of detection probability in the models we used is particularly important in estimating species richness of communities that contain a preponderance of rare, or difficult to detect, species [44]. In these cases, using traditional approaches may yield incorrect inferences if heterogeneity in detectability exists among species or if the effects of environmental covariates on occurrence differ among species.

In terms of species composition, the pool of ten most-detected species (>20 events) reveals the relative high occurrence of a number of species that are poorly known, and poorly detected using alternative methods. For example, the Abbott’s duiker is a IUCN-Endangered ‘giant’ duiker endemic to and found only in a handful of montane forests in Tanzania, including the Udzungwa population considered to be the stronghold, and was the third most common species in terms of occupancy, *ψ* = 0.72 [28], [45]. Similarly, the fourth most common Sanje mangabey (*ψ* = 0.62) is a predominantly terrestrial forest monkey endemic to only two forests in the Udzungwa mountains and classified as Endangered [28]. Being terrestrial and elusive, this monkey is poorly sighted from line-transects despite living in large groups of up to 50 individuals [46], and hence it is so far regarded as rarer than our data reveal. Among other commonly detected species, the relatively high ranking of tree hyrax (*ψ* = 0.48) is also surprising given this is known as an arboreal dweller [47]. Our data show that tree hyraxes spend more time on the ground than previously thought. It is also worth mentioning the 11^th^ position in the occupancy ranking of the Udzungwa-endemic and IUCN Vulnerable grey-faced sengi (or elephant-shrew), a species described in 2008 which is very rarely seen despite being diurnal, and for which Mwanihana holds approximately half of the global population [48]. The pool of least-detected species contains a number of truly arboreal mammals, typically the two colobine monkeys that are common in high densities across the forest [46], which are completely explained by their habit. Besides these ‘exceptions’, the other least-detected species are a diverse suite of less common (e.g. bush pig, Lowe’s servaline genet), or rare animals for the target forest (e.g. leopard, marsh mongoose), in addition to species that are mainly found in savannah and/or in the deciduous woodland occurring at the lower edge of Mwanihana forest (e.g. yellow baboon, African civet, banded mongoose, honey badger).

It is not surprising that we did not find any significant pattern of variation of estimated species richness across camera trap sites because Mwanihana forest has continuous forest cover without drastic habitat changes, except for the gradual variation in habitat type that broadly follows altitudinal and edge versus interior gradients. Whilst the species-specific occupancy models do highlight clear patterns of ecological preference by a suite of species, these preferences do not hold across the whole community. Interestingly, we found that the trophic guilds have significantly different detection probabilities. The low detectability of carnivores and insectivores matches their generally greater elusiveness relative to omnivores and herbivores. In contrast to our expectations, detection probability of species decreases with body mass, although the relationship is marginally significant (Table 3). Previous studies examining the effect of body mass on the animal detection process by camera traps suggest that small species are more likely to be missed due to the sensitivity and dimensions of the detection zone of the camera sensor [33], [49]. However, this aspect did not appear to have a statistical effect within the range of body mass in our study, perhaps because of the high sensitivity of the camera model we used. The relationship we found may rather reflect inter-specific behavioural differences, with larger species being less detected because of their greater elusiveness.

The species-specific occupancy analysis generally revealed novel ecological knowledge for roughly half of the species included in the analysis, excluding the strictly arboreal ones and those that are not typical forest-dwellers (see considerations above). The need to include corrections for imperfect detection in the modelling process is clearly shown by the remarkable variation of *p* among species (range 0.096–0.505; see Table 4). Because of this variation, the difference between naïve and estimated occupancy is also varying, and for the least-detectable species (*p*<0.2), the increment between naïve and estimated occupancy is 54–109% the naïve occupancy (see Table 4). The importance of allowing *ψ* and *p* to vary with covariates is shown by the fact that the null model was the least supported for any species. This is shown by a number of previous studies that investigated habitat associations from camera trapping data in an occupancy framework [21], [22], [36], [50]. To achieve similar inference for the remaining half of the species (*p*<0.1), a large number of sites should be surveyed [44]. Alternatively, one could pool data for more than one season under the assumption of a closed community (e.g. occupancy status does not change among survey seasons [22]).

The four limiting cases we highlighted show the particularly relevant ecological and conservation implications of our approach. For example, ecological knowledge on the Sanje mangabey was limited to results from a single, long-term focal group study located in the lower part of the forest [46], [51] before our analyses. There also was a lack of general understanding of their occurrence across the entire forest, which includes about half of the global population. Our results indicate that the species’ occupancy in montane forest is more than double than in lowland forest, which in turn suggests the vulnerability of this species to both human-induced (e.g. logging and forest degradation), stochastic (e.g. fires) and climate change impacts. The limit of using a categorical and broad classification of habitat type for this and other habitat-sensitive species may be overcome in future studies by collecting fine-scale vegetation and human disturbance data at camera trap sites for consideration in the modelling [22], [52]. Similar considerations apply to the results for the grey-faced sengi, whose preference for forest interior and edge avoidance matches the results from a recent focal study on habitat associations [22].

While the forest antelope community has been previously studied using camera trapping [29], [52] the fine grain occupancy models we derived shed new light into the occurrence of these species. Suni and Harvey’s duiker occur predominantly in the lower forest with the latter occurring across the forest edges. This is relevant to the need to protect the full array of forest cover, including the lower elevation areas, which border densely populated settlements. The preference of Harvey’s duiker for edges also indicates its suitability as an indicator of connectivity between forest blocks across marginal, often riverine habitat, which is important in highly heterogeneous areas such as the Udzungwas.

Despite a minority of species whose detection probability did not vary significantly with the covariates used (e.g. distance from edge and from border), the general finding is assuming that constant detection is broadly incorrect. Care needs to be taken when choosing covariates for *p* to ensure they are meaningful, which may be related to assumptions on the differences in the density of vegetation across camera trap sites. This assumption, in turn, may affect the efficiency of camera traps to capture an image of passing animals. In addition, variation in detectability may be due to differential animals’ shyness in relation to human disturbance and/or density of vegetation on the forest floor compressing the field of view of camera traps. These results may indicate a pattern of lower detectability in areas that are closer to human disturbance (e.g. border) and/or habitat ‘disturbance’ (e.g. border and edge), where forest floor vegetation is generally denser due to higher canopy degradation than in forest interior, and include animals that are more shy. The few cases of a negative relationship between *p* and one of the two covariates may also be explained by species-specific habits. For instance, the Sykes’ monkey’s *p* is negatively related to edge, which fits with the habit of this opportunistic primate to move easily among dense, degraded and regenerating vegetation in forest edge [30]. Similar considerations may also be valid for the bushy-tailed mongoose, a small, nocturnal and opportunistic carnivore that is often sighted by the park border and forest edge (FR unpublished data).

## Conclusions

Our study applied a robust analytical framework to profiling tropical mammal communities detected by the standard camera trapping protocol adopted by the TEAM Network. With the network currently made of 16 sites across three continents and progressively expanding (http://www.teamnetwork.org), and a number of studies adopting similar designs outside the network [36], there emerges a growing need for standardized analytical procedures to facilitate and enhance the sound use of the large data-sets being accumulated. In turn, detailed and site-specific baseline analysis will help interpreting patterns of community composition and changes from multi-site comparisons [8]. Similarly, with data collected from a number of sites for >5 years, baselines such as ours are relevant to the interpretation of temporal trends in species and community occupancy, for which robust and standardized analytical procedures have recently been proposed, including the Wildlife Picture Index [2], [14].

The ultimate relevance of standardizing tropical mammal community assessments rests in the need to develop indicators for distribution and abundance of pan-tropical species, as outlined by the Convention on Biological Diversity [2], [14]. In this context, our study offers an example of how analysis of species’ richness in occupancy framework, focal species’ occupancy and their spatial variation relative to a suite of covariates, represents a useful approach for comparing data from several sites, and hence for deriving indicators for these global targets.

## Supporting Information

Table S1 **Model selection details for the 11 species for which occupancy and detection probability were modelled with covariates.**
(DOCX)
