# Correction: Estimating Species Richness and Modelling Habitat Preferences of Tropical Forest Mammals from Camera Trap Data

**DOI:** 10.1371/journal.pone.0110971

**Published:** 2014-10-13

**Authors:** The PLOS ONE Staff

In the Acknowledgements section, the following information is missing: All data in this paper were provided by the TEAM Network, a partnership between Conservation International, The Missouri Botanical Garden, The Smithsonian Institution and The Wildlife Conservation Society.
