# Supplementary material for: Estimating Species Richness and Modelling Habitat Preferences of Tropical Forest Mammals from Camera Trap Data
Source: PLoS One. 2014 Jul 23;9(7):e103300. doi: 10.1371/journal.pone.0103300 (PMC4108438; doi:10.1371/journal.pone.0103300)
Supplement: Table S1 — Model selection details for the 11 species for which occupancy and detection probability were modelled with covariates. (DOCX) [file pone.0103300.s001.docx]

Table S.1. Parameter estimate (SE in parenthesis) of models of detection probability (*p*) and occupancy *(ψ)* with covariates for the 11 target species.

|  | **Border (*p*)** | | **Edge (*p*)** | |  | | **Border (*ψ*)** | | **Edge (*ψ*)** | | **River (*ψ*)** | | **Habitat (*ψ*)** | | **Slope (*ψ*)** | |  |
| --- | --- | --- | --- | --- | --- | --- | --- | --- | --- | --- | --- | --- | --- | --- | --- | --- | --- |
| *Cephalophus harveyi* | | 0.188 (0.11) # | | -0.155(0.12) | |  | |  | | -1.210(0.60)* | | 0.943(0.56)# | |  | |  | |
| *Bdeogale crassicauda* | | -0.256(0.12)* | | -1.184(0.14) | |  | | -5.584(14.8) | |  | | -19.37(31.0) | |  | | 29.87(47.6) | |
| *Cricetomys gambianus* | |  | |  | |  | | -0.580(0.65) | | -0.363(0.47) | | 0.998(0.44)* | | 1.474(1.47) | |  | |
| *Cercocebus sanjei* | | -0.278(0.22) | | -0.283(0.18) | |  | |  | |  | |  | | 1.804(0.78)* | |  | |
| *Cephalophus spadix* | | 0.858(0.23)*** | | 0.772(0.21)*** | |  | |  | |  | |  | |  | |  | |
| *Nesotragus moschatus* | |  | | 0.503(0.22)* | |  | | 0.911(0.49)# | |  | |  | | 3.130(1.06)** | |  | |
| *Paraxerus vexillarius* | |  | |  | |  | |  | | 1.293(0.49)** | |  | |  | |  | |
| *Rhynchocyon udzungwensis* | |  | |  | |  | | 0.619(0.56) | | 0.730(0.41)# | |  | | 2.109(1.24)# | |  | |
| *Cercopithecus mitis* | |  | | -0.804(0.37)* | |  | |  | |  | |  | |  | | 0.860(0.60) | |
| *Dendrohyrax arboreus* | |  | | 0.579(0.315)# | |  | |  | |  | | 2.831(1.35)* | |  | |  | |
| *Potamochoerus larvatus* | | -0.649(0.33)* | | -0.365(0.40) | |  | |  | |  | |  | |  | |  | |
| # 0.1< P <0.05 |  | |  | |  | |  | |  | |  | |  | |  | |  |
| * 0.05<P<0.01 |  | |  | |  | |  | |  | |  | |  | |  | |  |
| ** 0.01<P<0.001 |  | |  | |  | |  | |  | |  | |  | |  | |  |
| *** P<0.001 |  | |  | |  | |  | |  | |  | |  | |  | |  |
